# Tips and Tricks to Perform a Successful Vein of Marshall Alcohol Ablation

**DOI:** 10.19102/icrm.2025.16092

**Published:** 2025-09-15

**Authors:** Aashish Katapadi, Mahmoud Gomaa, Rayyan Bhutta, Aleena Arif, Emile Daoud, Muhammad R. Afzal

**Affiliations:** 1 Kansas City Heart Rhythm Institute, Overland Park, KS, USA; 2 Division of Cardiovascular Medicine, The Ohio State University Wexner Medical Center, Columbus, OH, USA; 3 TriHealth Heart & Vascular Institute, Cincinnati, OH, USA

**Keywords:** Alcohol ablation, atrial fibrillation, catheter ablation, vein of Marshall

## Abstract

Marshall bundle ablation via retrograde ethanol infusion into the vein of Marshall (VoM) is one of the few adjunctive approaches complementary to the success of pulmonary vein (PV) isolation during catheter ablation for persistent atrial fibrillation (AF). VoM ablation also increases the success and durability of mitral isthmus block for the management of peri-mitral flutter. Despite its promise, the adoption of VoM ablation is limited due to anatomical variations that result in a steep learning curve. Successful Marshall bundle ablation requires accurate identification and successful cannulation of the VoM with an appropriate-size balloon to achieve adequate occlusion, followed by non-traumatic ethanol infusion. VoM ablation is often performed before wide-area circumferential ablation of the left-sided PVs. Mitral isthmus ablation to achieve mitral annular block is always recommended after VoM ablation to minimize the risk of peri-mitral flutter. This paper discusses a step-by-step approach for successful Marshall bundle ablation with tips and tricks for difficult cases based upon the performance of over 500 cases performed at the Ohio State University Medical Center.

## Introduction

Atrial fibrillation (AF) affects 1%–2% of the global population, with a prevalence expected to more than double by 2050, and is associated with heart failure, thromboembolic stroke, earlier dementia and mortality, and an additional impact on a patient’s quality of life.^1^ Thus, treatment of AF is crucial. The 2023 American College of Cardiology/American Heart Association/American College of Clinical Pharmacy/Heart Rhythm Society Guidelines for the Diagnosis and Management of AF recommend rhythm control with anti-arrhythmic medications or catheter ablation (CA).^2^ Although the decision between the two is individualized through a shared decision-making process, multiple studies suggest that the latter is superior, and pulmonary vein (PV) isolation (PVI) is often recommended as a first-line therapy.^3^ Yet, the success rate of CA with durable PVI ranges from approximately 50%–80%, depending on several factors.^4^ Atrial tachycardia (AT) may also develop post-procedurally.

Beyond PVI, the only proven strategies to complement the success of PVI are Marshall bundle ablation via retrograde ethanol infusion into the vein of Marshall (VoM) and hybrid convergent ablation.^5,6^ The former was historically reserved for peri-mitral flutter. However, VoM ablation as an adjunctive strategy for persistent AF was found to be complementary to PVI. The Vein of Marshall Ethanol for Untreated Persistent Atrial Fibrillation (VENUS) trial and recently published Pulmonary Vein Isolation with Optimized Linear Ablation vs. Pulmonary Vein Isolation Alone for Persistent Atrial Fibrillation (PROMPT-AF) trial found that concurrent VoM ablation increases arrhythmia-free survival.^5,7^ Despite the promise of VoM ablation, this strategy has not been widely adopted due to a steep learning curve, procedural challenges, increased procedural time and cost, and lack of standardized criteria to define successful ablation. Consequently, this review discusses the most recent evidence, procedural approach, challenges associated with VoM ablation, and varied techniques to successfully achieve VoM ablation.

## Anatomy

**Supplementary Figure 1:**
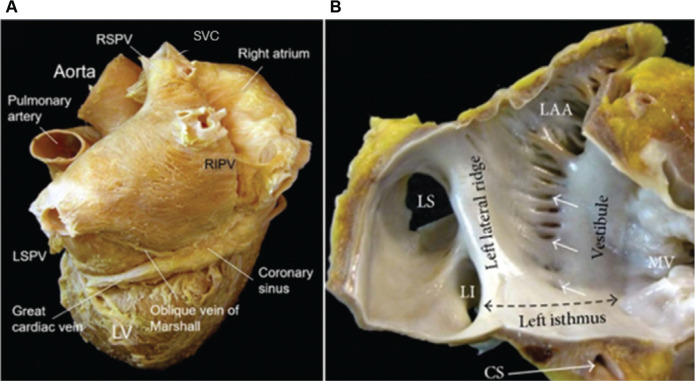
Anatomy of the vein of Marshall. The vein of Marshall branches off the coronary sinus **(A)** and is located within the mitral isthmus at the lateral ridge between the left atrial appendage and left pulmonary vein **(B)**. Republished with permission from Cabrera JA, Sánchez-Quintana D. Cardiac anatomy: what the electrophysiologist needs to know. *Heart*. 2013;99(6):417–431. *Abbreviations:* CS, coronary sinus; LAA, left atrial appendage; LI, left superior [pulmonary vein]; LS, left superior [pulmonary vein]; LSPV, left superior pulmonary vein; LV, left ventricle; MV, mitral valve; PV, pulmonary vein; RSPV, right superior pulmonary vein; SVC, superior vena cava; VoM, vein of Marshall.

The VoM, also known as the left atrial oblique vein, was first described by Marshall in the 1850s.^8^ It is a patent remnant of the left anterior cardinal vein (left superior vena cava), formed during the embryonic development of a right-sided venous system. It drains the posterior and posterolateral left atrium into the coronary sinus (CS). This development also creates the ligament of Marshall (LoM), within which the VoM is located **(Supplementary Figure 1)**.^9^ More specifically, the VoM is found within the mitral isthmus at the lateral ridge between the left atrial appendage (LAA) and the left-sided PVs. It is adjacent to the Marshall bundle, which is a muscular band that connects the LoM to the atrial myocardium and serves as an electroanatomic pathway between the left lateral ridge and the CS. The VoM also lies adjacent to and contains numerous sympathetic and parasympathetic innervations, being densest near the left atrial and PV junction and CS, respectively.^10^

## Background and efficacy

Stimulation of the left cardiac sympathetic nerve was initially found to generate an ectopic beat originating at the LoM/VoM.^11^ However, it was not until many years later that the role of the LoM was demonstrated in AF/AT and recognized as a potential therapeutic target.^12,13^ Shortly afterward, its role in autonomic mediation was confirmed.^14–16^ The first attempts at VoM ablation were conducted by Hwang et al., during which they recorded potentials within the VoM and successfully terminated AF by CA at its insertion site.^17^ Continued attempts at CA of the VoM were found to be technically challenging due to its anatomical location.^18^ As such, Valderrábano et al. proposed the use of ethanol infusion into VoM for a safe and effective method for ablation.^19^ In a first-in-human trial, they successfully ablated the VoM, inferior to the left inferior PV, in five out of six patients using two applications of 1 mL of 100% ethanol 2 min apart, with subsequent freedom from arrhythmia; one patient had recurrent atrial flutter, requiring ablation of the right superior PV. The effectiveness of this technique was further demonstrated for focal non–re-entrant and macro–re-entrant ATs, including those from the mitral annulus.^20^

Multiple studies have now established the role of adjunctive VoM ablation for AF patients^21^ **(Tables 1 and 2)**. The VENUS trial remains seminal, demonstrating that ethanol ablation at the VoM improved AF/AT freedom at 6 and 12 months (Δ = −11.2%; 95% confidence interval [CI], 0.8%–21.7%; *P* = .04).^5^ Another study where VoM ablation was followed by applications of radiofrequency energy targeting the bilateral PV antrum, cavotricuspid isthmus, and mitral isthmus reported even greater AF/AT freedom at 12 months (87.9% vs. 64.8%; *P* < .001) with a lower recurrence rate (hazard ratio, 0.27; 95% CI, 0.12–0.59).^22^ The Marshall-PLAN study found that the addition of VoM ablation and roof line to PVI in patients with persistent AF further improved AF/AT freedom.^23^ Meta-analyses demonstrated a lower recurrence rate (relative risk [RR], 0.58; 95% CI, 0.35–0.96; *P* = .04) and greater long-term freedom from AT/AF (RR, 1.28; 95% CI, 1.12–1.47; *P* < .001) when ablating the VoM compared to CA of PVI alone.^24,25^ Lastly, the recently published PROMPT-AF trial showed that linear and VoM ablation in addition to PVI significantly improved freedom from atrial arrhythmias within 12 months compared to PVI alone in patients with persistent AF.^7^

**Table 1:** Summary of Previously Published Studies Assessing for Vein of Marshall Ablation

Study	Year of Publication	Cont Group Int	VoM Group Int	Number of Patients	Type of AF
VENUS^5^	2020	RF for PVI + additional	EIVOM + RF for PVI + additional	343 total Int: 185 Cont: 158	Persistent
PROMPT-AF^7^	2024	RF for PVI	EIVOM + RF for PVI + linear ablation	498 (495 in analysis) Int: 246 Cont: 249	Longstanding persistent
Lai et al.^22^	2021	RF for PVAI + roof line + CTI + MI	EIVOM + RF for PVAI + roof line + CTI + MI	191 Int: 66 Cont: 125	Persistent
Okishige et al.^36^	2020	RF or CB only	RF or CB + EIVOM	342 (total with Cont) Int: 80 (PVI + EIVOM); 52 (CB + EIVOM) Cont: 90 RF; 120 CB	Paroxysmal
Takigawa et al.^37^	2020	RF at MI	EIVOM + RF for MI	103 Int: 32 Cont: 71	Post-AF AT
Ishimura et al.^38^	2021	RF for MI	EIVOM + RF for MI	560 Int: 176 Cont: 384 (including 301 only MI + 83 non-amenable to VoM)	Non-paroxysmal
Nakashima et al.^39^	2020	RF for PVI + MI	EIVOM + RF for PVI + MI	262 Int: 152 Cont: 110	Persistent
Gao et al.^40^	2022	RF for PVI + MI	76 total RF first, then EIVOM; EIVOM first, then RF for PVI + MI	165 total Int: 76: 28 (RF + EIVOM); 48 (EIVOM + RF) Cont: 89	AT
Ishimura et al.^41^	2023	RF for LAPW + MI	EIVOM + RF for LAPW + MI	413 Total Int: 177 Cont: 236	Paroxysmal and non-paroxysmal AF, AT
Shimizu et al.^42^	2023	RF for PVI + linear ablation (MI + roof line + CTI)	EIVOM + RF for PVI + linear ablation (MI + roof line + CTI)	233 total Int: 174 Cont: 50	Persistent

*Abbreviations:* AF, atrial fibrillation; AT, atrial tachycardia; CB, cryoballoon; Cont, control [group]; CTI, cavotricuspid isthmus ablation; EIVOM, ethanol infusion of vein of Marshall; Int, intervention [group]; LAPW, left atrial posterior wall; MI, mitral isthmus; PVAI, pulmonary vein antral isolation; PVI, pulmonary vein isolation; RF, radiofrequency.

**Table 2:** Procedural Characteristics and Outcomes of Previously Published Studies

VoM Cannulation Success	VoM Success %	Mitral Isthmus Block Success	12 Months Freedom of AF Success	Complications (Pericardial Effusion)	Average Procedure Duration (min)	Fluoroscopy Time if Given (min)
87.8% (216)	85% (209)	87.4% (15)	Int: 70.7% Cont: 61.5%	Int: 2 tamponade + 7 w/o drainage Cont: 1 tamponade	Int: 188 ± 54.1 Cont: 140.8 ± 39.7	Int: 15.9 ± 26.3 Cont: 5.1 ± 5.9
88.6% (117)	100% (117)	7.7% (10)	Int: 63.8% in RF + EIVOM; 82.7% in contact force + EIVOM	Int: 1 phrenic nerve + 1 cardiac tamponade + 3 pericardial effusion without int + 23 pericardial effusion + 2 CS dissection Cont: 0 phrenic nerve + 3 cardiac tamponade + 1 pericardial effusion w/o int; 23 pericardial effusion	Int: 45 ± 15 (PVI) + 17.8 ± 5.6 (EIVOM) Cont: 48 ± 15	
			Int: 72.2% Cont: 59.2%	Int: 1 pericardial effusion Cont: 5 pericardial effusion	Int: 248 (240–274) Cont: 300 (240–335)	Int: 71.5 (55.3–82) Cont: 61 (50–88)
	100% (133)	96% (128)		Int: 0 pericardial effusion Cont: 4 pericardial effusion	Int: 181 ± 47 Cont: 170 ± 54	
96.8% (152/157 planned)		98.7% (150)	Int: 68.4% Cont: 75.5%	Int: 0 Cont: 1 cardiac tamponade	Int: 276 ± 60 (PVI) + 21 ± 13 (EIVOM) Cont: 263 ± 69	Int: 384 (211–621) Cont: 344 (175–519)
81.8% (54/66)	80.3% (53)	95.5% (63)	Int: 87.9% Cont: 64.8%	Int: 1 mild pericardial effusion + 1 “fluid overload” Cont: 1 pericardial effusion + 2 AV fistula + 1 pleural effusion + 4 “fluid overload”	Int: 162 ± 39.7 Cont: 171.5 ± 44.8	Int: 11.8 ± 8.3 Cont: 4.3 ± 3.7
	RF-EIVOM: 42.8% EIVOM-RF: 60.4%	RF-EIVOM: 89.3% EIVOM-RF: 95.8%	Int: 79.6% (RF-EIVOM); 75.0% (EIVOM-RF) Cont: 56.2%	Int: 2 pericardial effusion w/o int; 3 (1 RF-EIVOM and 2 EIVOM-RF) CS dissection; 2 pericarditis Cont: 0	Int: EIVOM-RF: 164 ± 42 (RF-EIVOM); 154 ± 59 (EIVOM-RF) Cont: 133 ± 52	Int: 13.6 ± 11.0 (RF-EIVOM); 12.7 ± 8.0 (EIVOM-RF) Cont: 6.8 ± 7.8
43% (177/413; EIVOM attempted in all patients)		92% (≥5 mL ETOH); 96% (≤5 mL ETOH)	Int: 53% (≥5 mL ETOH); 53% (<5 mL ETOH) Cont: 66%	Int: 1 pericardial effusion Cont: 1 pericardial effusion	Int: 200 ± 53 (≥5 mL ETOH); 178 ± 43 (<5 mL ETOH) Cont: 154 ± 52	Int: 90 ± 31 (≥5 mL ETOH); 70 ± 25 (<5 mL ETOH) Cont: 66 ± 32
Not listed. Noted from successful VoM infusion. 83.8% (155/185)		80.6% (125)	Int: 49.2% Cont: 38%	Int: 1 pericardial effusion + 2 pericardial effusion requiring drainage + 10 pericardial effusion not requiring drainage + 3 hematoma + 1 CVA + 1 TIA + 7 fluid overload + 2 pneumonia + 3 death Cont: 1 pericardial effusion + 2 pericardial effusion requiring drainage + 6 pericardial effusion not requiring drainage + 6 hematoma + 2 pseudoaneurysm + 2 CVA + 2 TIA + 2 fluid overload + 4 pneumonia + 2 death	Int: 215.9 ± 77.7 Cont: 190.3 ± 63.5	Int: 23.0 ± 32.7 (VoM + RF) Cont: 11.9 ± 21.2
		96.2% (50/52)	Int: 82.0% (at 1026 ± 467 days) Cont: 64.9%	Int: 2 VoM dissection Cont: 2 pericardial effusion		

*Abbreviations:* AF, atrial fibrillation; AV, atrioventricular; CS, coronary sinus; CVA, cerebrovascular accident; EIVOM, ethanol infusion of vein of Marshall; PVI, pulmonary vein isolation; RF, radiofrequency; TIA, transient ischemic attack; VoM, vein of Marshall.

## Approach to ethanol infusion for vein of Marshall ablation

Multiple studies have reported varying protocols for VoM ablation, and, in this paper, the authors describe their protocol and strategies.^26^ Despite differences in approach, VoM ablation has been reported to be successful in up to 90% of cases.^27^ The authors typically follow a workflow where VoM ablation is performed first, followed by left-sided PVI, endocardial mitral isthmus ablation, right-sided PVI, and—if required—epicardial mitral isthmus ablation. Previous studies have shown that performing VoM ablation first increases success and minimizes the need for endocardial ablation in patients undergoing mitral isthmus ablation for peri-mitral flutter.^28^ Additionally, almost half of patients demonstrate isolation of the left inferior PV after VoM ablation (personal communication and authors’ experience). A typical VoM ablation workflow involves the following steps.

### Coronary sinus access with a steerable sheath

**Figure 1:**
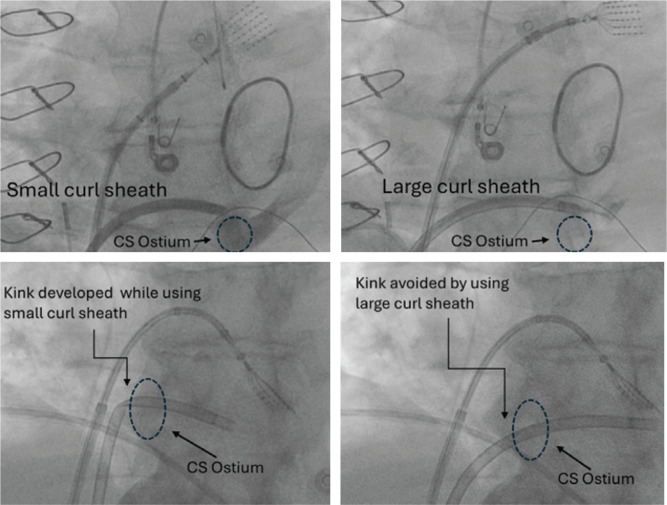
Cannulation of the coronary sinus (CS) with variable curl-sized steerable sheaths. The upper panels show the variable reach of the small curl (left) and large curl (right) sheaths to the ostium of the CS. The bottom panel shows that a small curl sheath (left) develops a kink once advanced into the CS. The kink is avoided when a large curl sheath (right) is used. *Abbreviation:* CS, coronary sinus.

Ablation of the VoM starts with successful cannulation of the CS. This can be achieved via the internal jugular or femoral veins. Both approaches are safe and efficacious; however, the femoral approach is becoming increasingly common and aligns with the standard workflow of AF ablation. The techniques described in this paper assume a femoral approach. After advancing a steerable sheath (Agilis NxT; Abbott, Chicago, IL, USA) into the right atrium, the CS is cannulated with a steerable catheter (decapolar catheter; Biosense Webster, Diamond Bar, CA, USA). Once stabilized in the body of the CS, the catheter is used as a rail to advance the steerable sheath into the CS. Previous studies have reported a lack of CS access in approximately 5% of cases due to variable CS anatomy. In the authors’ experience, unsuccessful CS cannulation rates are <1%. The most common reason for difficult CS cannulation is an enlarged right atrium or the presence of a prominent Thebesian valve. A large curled steerable sheath facilitates entry into the CS in patients with an enlarged right atrium and/or a prominent Thebesian valve **(Figure 1)**. Additionally, in patients with a sizable Thebesian valve and sub-Thebesian pouch, contrast injection at the interatrial septum can delineate the CS ostium for successful access. Visualization of the CS ostium with an intracardiac echocardiogram can minimize the need of fluoroscopy in difficult cases.

### Techniques to identify the vein of Marshall

**Figure 2:**
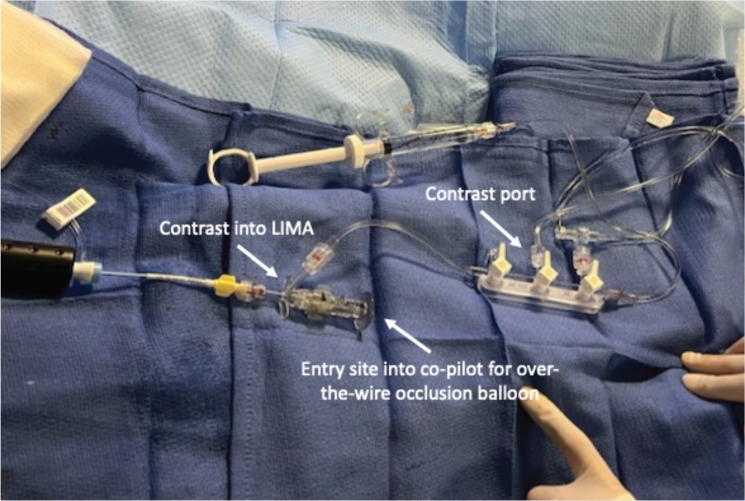
Manifold setup for the procedure. During this arrangement, a co-pilot is attached at the back end of the left internal mammary artery (LIMA) catheter. The manifold is attached to the side port on the co-pilot to allow intermittent contrast injections via the LIMA catheter to identify the vein of Marshall. The backend of the co-pilot provides access for over-the-wire occlusive balloon. *Abbreviation:* LIMA, left internal mammary artery.

**Figure 3:**
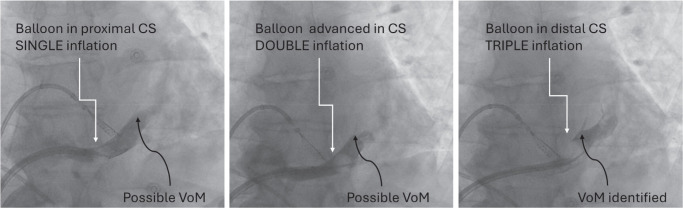
Occlusive venography of the coronary sinus (CS) to identify the vein of Marshall (VoM). The left panel shows CS venography when the balloon (white arrow) is in the proximal part of CS and is single-inflated (3 mL of air). Faint staining is noted in what appears to be the VoM. The middle panel shows that the balloon is double-inflated (two injections of 3 mL of air) and advanced into the middle portion of the CS. In the right panel, the balloon is triple-inflated (three injections of 3 mL of air) and further advanced into the CS, which makes the VoM obvious. *Abbreviation:* CS, coronary sinus.

Identification of VoM can be achieved by various techniques. The first is rather straightforward. With the tip of a 5-Fr left internal mammary artery (LIMA) catheter pointing toward the atrial side of the CS, intermittent injections of contrast can be used to help identify the VoM ostium and course. After the VoM is visualized, the LIMA catheter can be used for cannulation. The second technique to identify the VoM is with CS venography using an occlusive balloon.^29^ An appropriate setup using a manifold can facilitate contrast injections as needed **(Figure 2)**. The authors routinely advance a 105-cm-long 6-Fr CS balloon venography catheter over a straight 0.035-in wire (TigerWire; Abbott). Advancing the balloon over the 0.035-in wire avoids inadvertent trauma from the tip of the balloon. The balloon is typically inflated proximal to the valve of Vieussens (VoV), and the tiger wire is removed. The VoV can be identified in the left anterior oblique (LAO) view on occlusive CS angiography. The VoM drains proximal to the VoV. The distance of the VoV from the CS ostium can be variable. Due to the posterior and cranial course of the VoM, a right anterior oblique (RAO) view is most suitable for its identification. It is essential to realize that the VoM often has multiple small branches (which course posteriorly, in an RAO view at 10 and 11 o’clock positions). If the VoM is not identifiable despite an occlusive CS venography, then the balloon could be advanced in an RAO view until it buckles against the VoV. A contrast injection in the RAO view often identifies the VoM **(Figure 3)**.

### Differentiation of the vein of Marshall from other atrial veins

**Figure 4:**
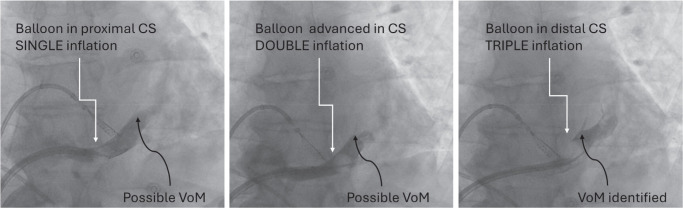
**A–C:** Differentiation of the vein of Marshall (VoM) from the left and right atrial veins. Once the occlusive balloon is inflated proximal to the valve of Vieussens, the VoM, left atrial appendage veins, and right atrial veins are visible. The atrial branches of the coronary sinus besides the VoM are better visualized in the right anterior oblique view **(B)** compared to the left anterior oblique view **(A)**. *Abbreviations:* LA, left atrial; LAA, left atrial appendage; PW, posterior wall; VoM, vein of Marshall.

The right atrial and LAA veins can also be identified in an RAO view. The former typically courses posteriorly toward a 9 o’clock position, whereas the LAA vein is distal to the VoM and courses straight up or slightly anteriorly toward a 1 o’clock direction **(Figure 4)**.

### Cannulation of the vein of Marshall with the left internal mammary artery catheter and 0.014-in wire

**Figure 5:**
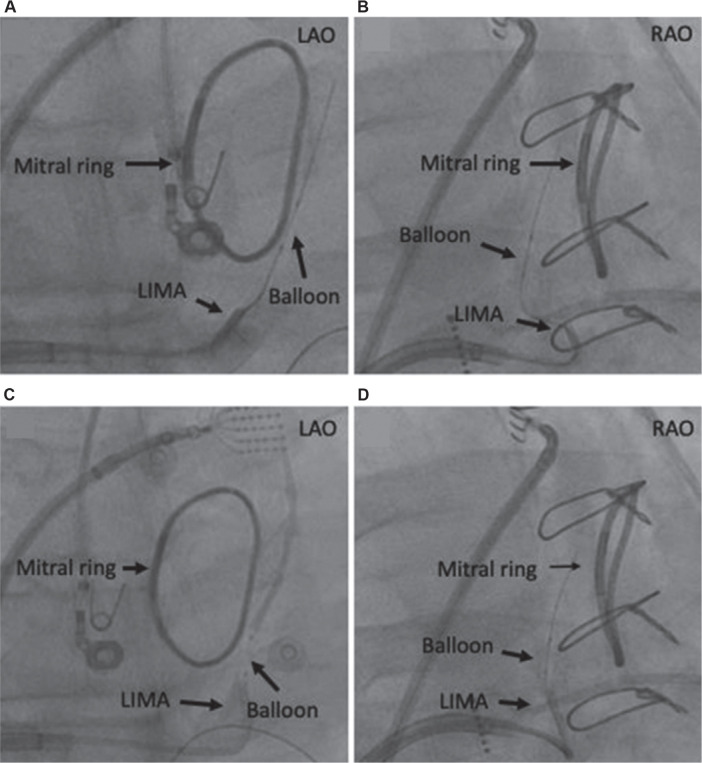
**A–D:** Multiple fluoroscopic views to ensure co-axial alignment of the left internal mammary artery (LIMA) and over-the-wire balloon. The upper panels show a lack of co-axial alignment of the LIMA and vein of Marshall balloon when viewed in different views. After advancing the LIMA catheter, both left and right anterior oblique views demonstrate co-axial alignment (lower panel). *Abbreviations:* LAO, left anterior oblique; LIMA, left internal mammary artery; RAO, right anterior oblique.

The VoM is most commonly cannulated with a 5-Fr LIMA guide catheter that is advanced through the steerable sheath over a 0.035-in wire. The 0.035-in wire is advanced beyond the VoV so that the LIMA catheter can be parked distal to the VoV to avoid inadvertent cannulation of the VoM. Inadvertent cannulation of the VoM with LIMA may potentially damage the VoM ostium and result in acute thrombosis, making subsequent cannulation with a coronary angioplasty balloon difficult. After the distal tip of the LIMA catheter is beyond the VoV, a coronary angioplasty balloon over a 0.014-in guidewire is advanced into the catheter. The selection of the diameter and length of the angioplasty balloon depends on the size of the VoM. The authors often use a 1.5 or 2.0 × 10-mm balloon. After advancing the angioplasty balloon and 0.014-in wire closer to the distal tip of the LIMA catheter, the LIMA catheter is brought adjacent to the VoM, guided by intermittent contrast injections via the manifold. The use of a manifold allows intermittent contrast injections while the VoM is being cannulated with a 0.014-in wire. Next, the 0.014-in wire is advanced into the VoM. Extreme caution should be exercised during this step to avoid advancing the 0.014-in wire into any smaller tributaries of the VoM. The perforation of smaller tributaries is a potential risk for all 0.0140-in wires and may result in preferential alcohol leakage outside the venous bed into the space between the myocardium and the visceral layer of the pericardial space. Once the 0.014-in wire is inside the main body of the VoM, the coronary balloon is advanced over the wire while slowly dragging the wire back to minimize forward entry of the wire into venous tributaries. The balloon is inflated once inside the VoM. After the balloon is inflated and “anchored,” the LIMA catheter is advanced to the ostium of the VoM. The LIMA catheter should be co-axial to the balloon to minimize kinking of the balloon. This can be confirmed with contrast injection through the LIMA catheter. Alternating RAO and LAO views can be helpful to ensure that the LIMA catheter is co-axial to the coronary balloon **(Figure 5)**. Once the balloon is noted to be stable and the LIMA catheter is at the VoM ostium, the coronary wire is removed.

### Vein of Marshall alcohol infusion strategy

During this procedure, the balloon is flushed using a 1-mL syringe, followed by contrast injection to visualize the venous bed. Initial hazy staining is often noted with contrast injection; bright staining usually indicates perforation of the venous system and entrapment of the contrast between the atrial tissue and visceral pericardium. Once an adequate seal of the VoM is noted, the first 3 mL of alcohol is injected over 1 min. The operators perform a contrast injection after every 3 mL of alcohol to assess for different characteristic staining patterns. Like the initial injections, diffuse hazy staining with a dotted appearance indicates adequate damage to the VoM tributaries. On rare occasions, contrast can be seen in the pericardial space and is often inconsequential. If the size of the VoM allows, the initial inflation of the balloon and injection should be in the distal part of the VoM. After 1–3 mL of ethanol infusion, the balloon can be deflated and brought back toward the VoM ostium. This allows alcohol entry into all tributaries of the VoM. The optimal volume of alcohol is not well studied in the published literature. Previous studies have variably used either 3 or 10 mL of total volume equally split into three separate injections. The authors routinely use 10 mL of alcohol as described in the largest single-center experience.^30^

### Left atrial appendage and left-sided pulmonary vein ridge echocardiography

**Supplementary Figure 2:**
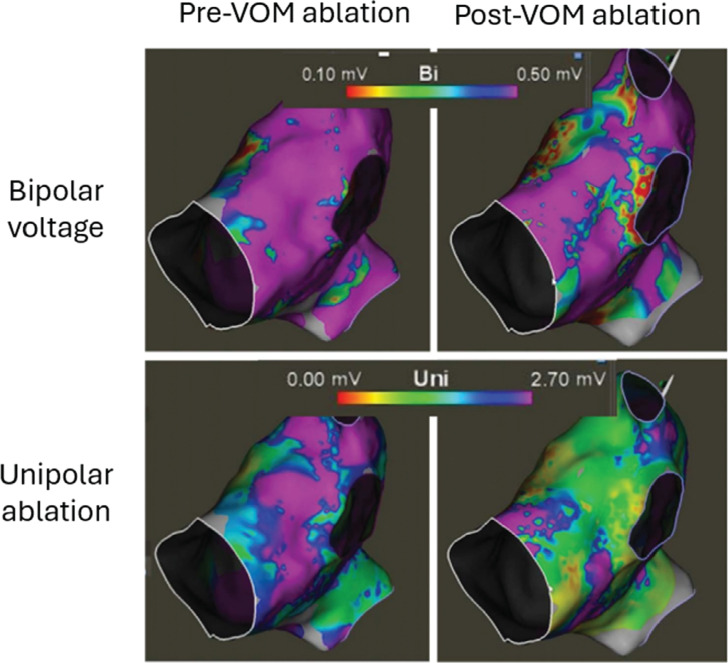
Impact on unipolar voltage loss after vein of Marshall (VoM) ethanol infusion. The upper panels show bipolar voltage before (left) and after (right) VoM ethanol infusion. The bottom panels show unipolar voltage before (left) and after (right) VoM ethanol infusion. *Abbreviation:* VoM, vein of Marshall.

Identifying and cannulating the VoM can be technically challenging. Even more difficult is ensuring that ethanol does not leak outside of the venous bed into the space between the myocardium and visceral pericardium. Intracardiac echocardiography (ICE) from the right ventricular outflow tract can identify the intense brightness at the ridge between the LAA and left-sided PVs, which often correlates with voltage loss between the left-sided PVs and the LAA **(Supplementary Figure 2)**. The presence of increased myocardial local echogenicity at the ridge and consistent echogenic streaming in the LA detected by ICE-based imaging during VoM ethanol infusion suggests increased ablated tissue in that region, as reported in a recent study.^31^

### Electroanatomic mapping

**Figure 6:**
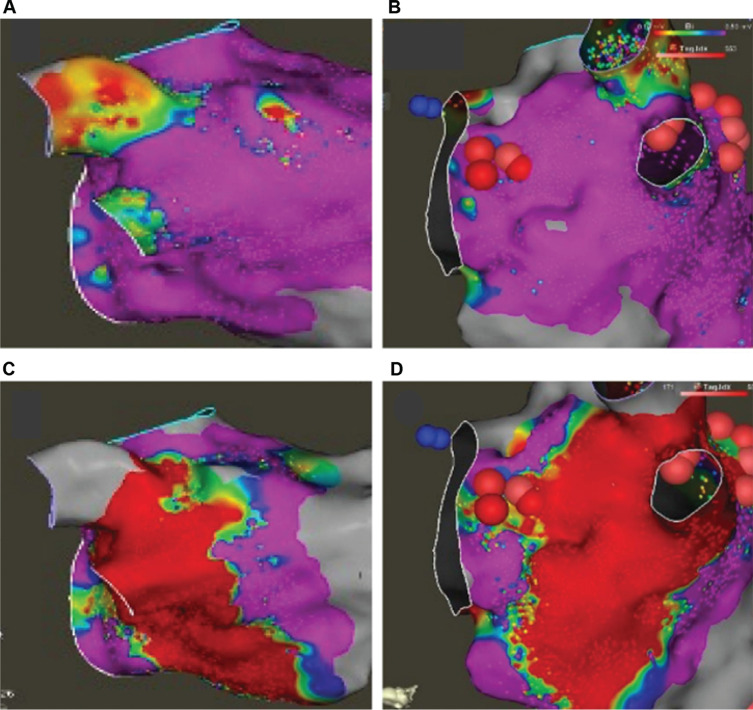
Electroanatomic map after vein of Marshall injection. The upper panels reveal baseline bipolar voltage around the left inferior pulmonary veins in posteroanterior **(A)** and left lateral **(B)** views. The bottom panels show characteristic voltage loss after vein of Marshall ethanol infusion in the corresponding orientation **(C, D)**.

**Supplementary Figure 3:**
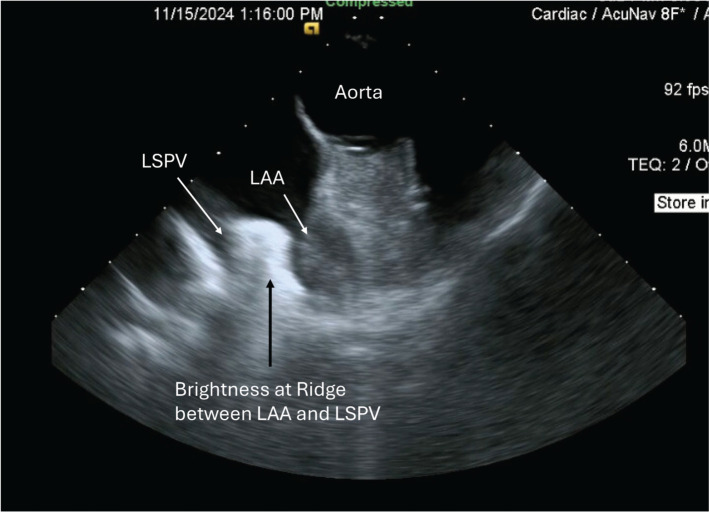
Intracardiac echocardiogram appearance after ethanol injection. Intracardiac echocardiogram short-axis view from the right ventricular outflow tract reveals the brightness at the ridge between the left atrial appendage and left superior pulmonary vein. *Abbreviations:* LAA, left atrial appendage; LSPV, left superior pulmonary vein.

Post-procedural, high-density electroanatomic mapping is performed to assess for characteristic voltage loss **(Figure 6)**. This is often performed directly after VoM ethanol infusion and before PVI. The most distinctive pattern reveals voltage loss under the left inferior PV in a strawberry pattern, but various patterns of voltage loss have been suggested.^32^ Significant damage is noted between the left inferior PV and mitral annulus, sparing a small amount of healthy tissue toward the latter side. At this point, wide-area circumferential ablation is performed on the left side. However, in our experience, the left inferior PV is isolated with VoM injection alone in over 60% of cases, and often minimal ablation is needed to achieve isolation of the left-sided PVs. Often, after successful VoM injection, voltage loss is not noted. Due to the presence of tissue damage in the epicardial aspect, unipolar voltage may often reveal voltage loss in the characteristic area **(Supplementary Figure 3)**. However, this observation has not been systematically studied or described. The loss of voltage was previously reported to increase progressively.^33^ In the authors’ experience of repeat mapping for redo ablations in patients with previous VoM injections, the voltage loss was noted to be persistent.

### Mitral annular block after the vein of Marshall injection

After successful VoM ablation, small amounts of atrial tissue between the left PV and mitral annulus are preserved and often act as an isthmus for peri-mitral flutter. Thus, it is recommended that a mitral isthmus block be performed after VoM ablation. A multipolar catheter is advanced into the LAA for pacing, and CS activation is assessed. In the presence of conduction through the mitral isthmus, CS activation is distal to proximal. At this point, endocardial ablation between the mitral annulus and left inferior PV is performed. Once the mitral isthmus block is achieved, the CS activation reverses from proximal to distal, indicating a successful block. Any residual gaps in the annular aspect of the mitral isthmus or CS are addressed with additional ablation. In approximately a third of the cases, ablation within the CS, overlapping the location of the endocardial ablation lesions, is required to achieve mitral annular block. For successful ablation, the ablation catheter must be advanced to the distal aspect of the CS, beyond the line of endocardial ablation for the mitral isthmus. The lesions are delivered just superior, with the catheter facing toward the ventricular side. This is indicated by larger ventricular signals. If this does not achieve block, then a circular ablation in the CS targets any remaining connections.

### Discordant endocardial and epicardial coronary sinus activation

**Figure 7:**
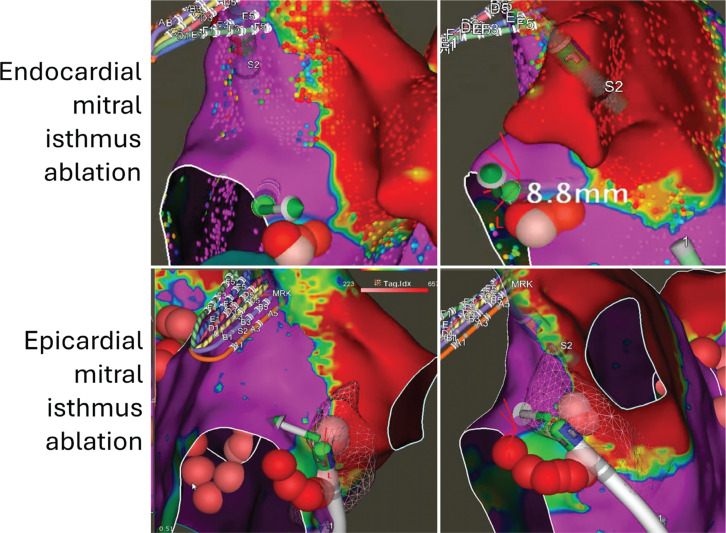
Endocardial and epicardial ablation to achieve mitral isthmus ablation. The upper panels show the location of the ablation catheter in the endocardial aspect of the posterior mitral isthmus line. The force vector should point toward the atrial tissue. The lower panels show the presence of the ablation catheter in the coronary sinus (mesh). The force vector is pointing toward the atrial tissue.

**Figure 8:**
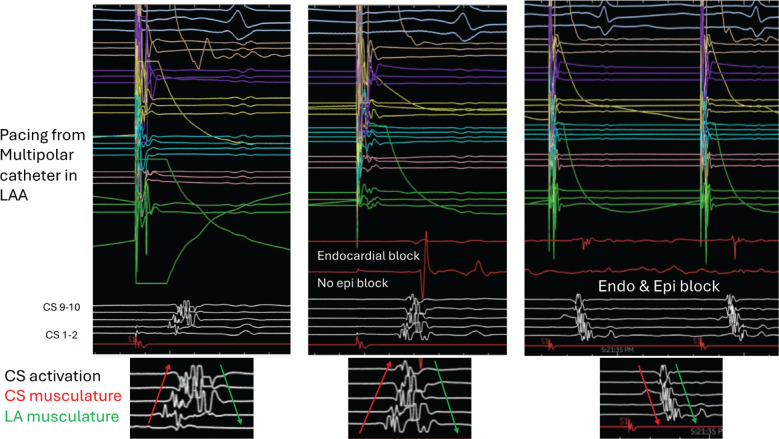
Assessment of mitral isthmus block. The upper part (colored signals) of each panel shows pacing from the multipolar catheter (Optrell) located in the left atrial appendage and coronary sinus (CS), and the lower part and the enlarged insert (white signals) show CS activation (white). The CS activation pattern in the left panel was noted after vein of Marshall ablation. The CS signals reveal two distinctly different patterns. The near-field sharp signals (CS musculature) show distal to proximal activation (lack of mitral isthmus block), while far-field signals (left atrial muscular) show proximal to distal activation (presence of mitral isthmus block). The middle panel shows the presence of endocardial mitral isthmus block, as shown by delayed ablation signals compared to distal CS epicardial signals. The right panel shows a complete reversal of CS activation, indicating endocardial and epicardial block. *Abbreviations:* CS, coronary sinus; LA, left atrial; LAA, left atrial appendage.

Successful mitral isthmus ablation is recommended after VoM ablation. Previous studies have shown a failure rate of 10%–15% despite successful VoM ablation. Mitral isthmus ablation after successful VoM ablation is achieved by performing endocardial ablation often at the ventricular aspect of the mitral isthmus. CS ablation is needed in 40%–60% of cases in the authors’ experience **(Figure 7)**. For successful CS ablation, the catheter must be advanced past the endocardial line of ablation at the mitral isthmus. The lesions are delivered just superior, with the catheter facing toward the atria and/or ventricular side. Successful mitral isthmus block is confirmed by assessing CS activation during pacing from the LAA. In the absence of mitral isthmus block, the CS activation sequence is distal to proximal. With mitral isthmus block, the activation pattern changes to proximal to distal. Often, there is discordant endocardial and epicardial CS activation where CS signals reveal two distinctly different patterns **(Figure 8)**. In the given example, the near-field sharp signals (CS musculature) show distal to proximal activation, indicating the absence of epicardial mitral isthmus block, while far-field signals (left atrial musculature) show proximal to distal activation, indicating the presence of endocardial mitral isthmus block.

### Post-procedural care

Successful VoM ablation results in a significant amount of tissue damage with ensuing intense inflammatory reaction and pericarditis. The authors often prescribe a 5-day course of oral steroids, which is sufficient for symptom control. If pericarditis symptoms persist after the completion of the steroid course, colchicine is used as an alternative, with a typical dose of 0.6 mg twice a day for 2 weeks. Some operators may prefer colchicine over a short course of prednisone.^34^

### Potential complications

Successful VoM alcohol ablation requires adequate operator experience for navigation in the CS and familiarity with coronary wires and balloons. There are various potential complications that can happen during sheath, catheter, and balloon manipulation in the CS. The earlier experience with VoM injection was primarily limited to patients with peri-mitral flutter, and the operators were not routinely performing the VoM ablation for AF. Therefore, the complication rate was higher in the early experience. With increasing experience of VoM alcohol ablation for AF, the incidence of complications is low. In the authors’ experience of >500 cases, only one patient developed pericardial effusion requiring pericardiocentesis. The other potential complication is isolation of the LAA if alcohol is inadvertently injected in the LAA branch. This can be minimized by adopting routine occlusive venography of CS for the identification of various venous tributaries of CS.

### Mitral isthmus ablation in the era of pulsed field ablation

Following the advent of pulsed field ablation (PFA), there has been an increasing interest in using PFA for mitral isthmus block. The initial data suggest that VoM ablation has better efficacy for mitral isthmus block compared to radiofrequency ablation.^35^

## Conclusion

VoM ethanol ablation can improve outcomes in patients with persistent AF and peri-mitral flutter. While this technique has yet to be widely adopted, electrophysiologists can easily learn it. Ethanol administration and procedural factors can be further personalized for increased efficacy. With the emergence of PFA, the role of VoM ablation to achieve transmural mitral isthmus ablation needs to be redefined.
